# Pars Distalis and Pars Tuberalis Thyroid-Stimulating Hormones and Their Roles in Macro-Thyroid-Stimulating Hormone Formation

**DOI:** 10.3390/ijms241411699

**Published:** 2023-07-20

**Authors:** Eleonore Fröhlich, Richard Wahl

**Affiliations:** 1 Center for Medical Research, Medical University of Graz, 8010 Graz, Austria; eleonore.froehlich@medunigraz.at; 2 Department for Diagnostic Laboratory Medicine, Institute for Clinical Chemistry and Pathobiochemistry, University Hospital Tübingen, 72076 Tübingen, Germany

**Keywords:** macro-hormones, thyroid-stimulating hormone, pars tuberalis TSH, pars distalis TSH, immunoassay, circadian changes, ultradian changes, seasonal changes

## Abstract

Thyroid-stimulating hormone (TSH) and thyroid hormone levels are standard parameters in blood analysis. However, the immunoassays employed may lead to false-positive or false-negative results when the sample contains certain materials that interfere with the assay. Macro-TSH, a complex of TSH with immunoglobulin or albumin, may cause apparently increased TSH concentrations. TSH is produced in the pars tuberalis (PT) of the pituitary gland and by thyrotrophs of the pars distalis (PD). It was found that variable glycosylation can render the molecule more strongly bound to antibodies or albumin in the blood, leading to the hypothesis that macro-TSH consists mainly of PT-TSH. Although less known than PD-TSH, PT-TSH plays an important role in the central regulation of thyroid metabolism. The present review summarizes the physiological function of human PT-TSH and its role in macro-TSH formation. The prevalence of macro-hyperthyrotropinemia, the structure of PT-TSH and macro-TSH, problems in the measurement of TSH, and the action of PT-TSH in animals with seasonal breeding are discussed. Despite the absence of a specific function of macro-TSH in the organism, the identification of macro-TSH is important for avoiding unnecessary treatment based on a falsified readout of increased TSH concentrations as numerous individual case reports describe.

## 1. Introduction

Thyroid-stimulating hormone (TSH) and thyroid hormone levels are standard parameters in blood analysis. The TSH level is the most important parameter to identify subclinical hypo- or hyperthyroidism. However, one problem for routine diagnosis is that the immunoassays commonly used to quantify TSH may generate false-positive or false-negative results when samples contain materials that interfere with the test or are incorrectly determined by this technology, like macro-TSH. Macro-TSH is defined as a large molecular-sized TSH that is mostly a complex of TSH and IgG or albumin. Patients with macro-TSH typically have elevated serum TSH and normal free thyroxine concentrations, mimicking subclinical hypothyroidism. The important feature of macro-TSH is that it mainly contains TSH produced by the pars tuberalis (PT) of the pituitary gland [1]. This molecule is generally less known than systemically active TSH produced by thyrotrophs of the pars distalis of the gland (PD). The aim of this review is, on the one hand, to highlight the different physiological roles of PT-TSH and PD-TSH and, on the other hand, to address problems in the identification of macro-TSH formation and in the measurement of TSH concentrations. For many researchers, macro-TSH represents merely a diagnostic problem. This review intends to provide more insight into the properties of its main component PT-TSH in comparison to PD-TSH.

## 2. The Two Sources of Pituitary TSH

**Figure 1:**
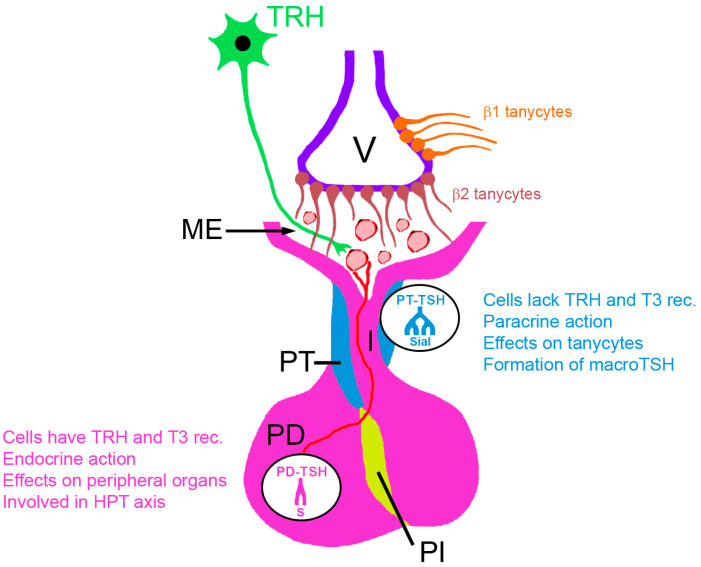
Regulation of TSH secretion. Hypothalamic neurons end with terminal buttons at the median eminence (ME) in the upper region of the infundibulum and secrete thyrotropin-releasing hormone (TRH) into the portal system of the pituitary gland. Via this portal system at the ME, TRH is transported to the pars distalis of the anterior pituitary gland (PD) and induces production and secretion of TSH. Pars tuberalis (PT), the source of PT-TSH, represents a thin sheath at the pituitary stalk. In rats, an independent mode of action on thyrotrophs of the PT was found. Melatonin directly inhibits TSH production from these thyrotrophs. This action reduces both the binding of PT-TSH to its receptor on tanycytes lining the 3rd ventricle (V) and the conversion of thyroxine (T4) to triiodothyronine (T3) by intracellular deiodinase type 2 (DiO2). In the systemic circulation PT-TSH is detectable as macro-TSH. PT-TSH and PD-TSH molecules differ in their glycosylation pattern. PD-TSH has biantennary sulfonated glycans, whereas macro-TSH has multi-branched, sialylated N-glycans. Abbreviations: PI, pars intermedia; PP, posterior pituitary lobe; Sial, sialylated glycans; S, sulfonated glycans.

TSH is the main stimulator of thyroid hormone production in the thyroid gland. It is produced in the pars distalis (PD) of the anterior pituitary gland and secretion has a circadian rhythm partly controlled by neurons and astrocytes of the suprachiasmatic nucleus (SCN), which influence the secretion of thyrotropin-releasing hormone (TRH) by the paraventricular neurons of the hypothalamus [2,3] (Figure 1). Neural projections from the SCN to the TRH-releasing neurons of the paraventricular nucleus induce the rhythmic TRH secretion, which induces in turn the release pattern of TSH in the thyrotrophs of the PD. Both secretion of TRH and of TSH are part of the hypothalamic–pituitary axis, and are inhibited by high blood concentrations of thyroid hormones, particularly of triiodothyronine (T3) [4]. TRH is produced by neurons of the paraventricular nucleus of the hypothalamus and released at the pituitary stalk into the pituitary portal system. Transported by the blood, TRH reaches the thyrotrophs of the PD of the anterior part of the pituitary gland, binds to TRH receptors and induces transcription of the TSH α and β subunit. TSH produced in the pars distalis, PD-TSH, binds to TSH receptors of the follicular cells of the thyroid gland.

A second site of synthesis of TSH is located in the PT. PT-TSH was identified in seasonal breeding mammals, where it plays an ancestral role in seasonal reproductive control of vertebrates [5]. The PT of the pituitary gland is located at the median eminence (ME), which, together with the area postrema, belong to the “circumventricular organs” [6]. In this region, capillary vessels lack the blood–brain barrier (BBB) and are fenestrated. Tanycytes control the release of TRH into the hypothalamo–pituitary portal system to stimulate thyrotrophs in the PD. In contrast to TSH from the PD, thyrotrophs of the PT lack TRH and thyroid hormone receptors and, therefore, are not involved in the feedback regulation of thyroid hormone levels [3]. PT-TSH, in contrast to PD-TSH, exerts local/paracrine action [7]. PT-TSH can reach the bloodstream via the fenestrated capillary vessels in the ME. Blood concentrations of PT-TSH are 10,000 times lower than those of PD-TSH [1].

**Figure 2:**
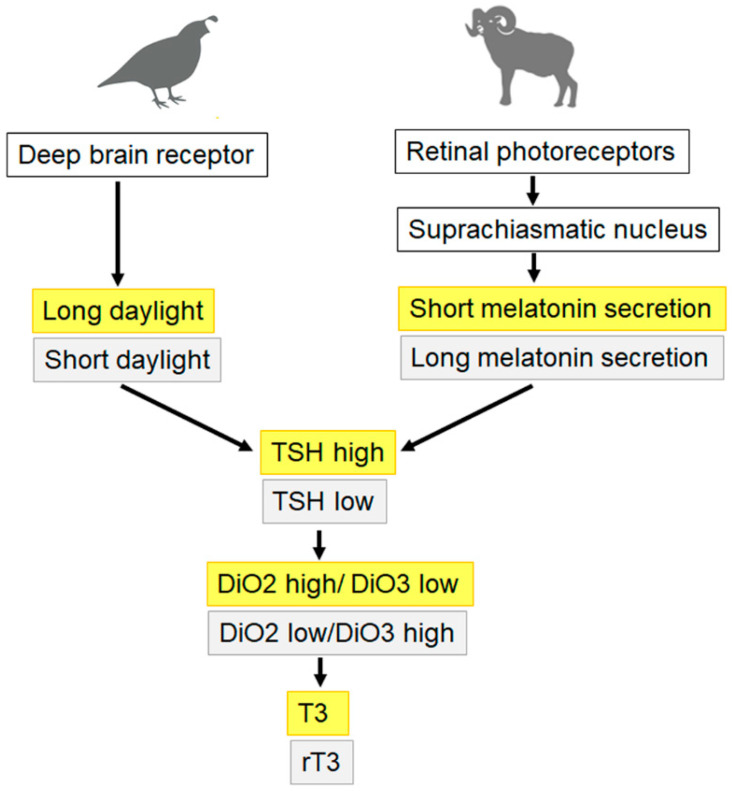
PT-TSH secretion in birds (e.g., Japanese quails, sparrow) is directly regulated by light via activating a receptor in the deep brain. Long daylight induces PT-TSH, increases deiodinase type 2 (DiO2) activity and generates-bioactive T3. Activation of DiO3 by short daylight leads to preferential conversion of T4 to the inactive metabolite reverse T3 (rT3). In mammals (e.g., sheep, goat, hamster, ground squirrel, common vole, Fisher 344 rats), retinal photoreceptors act on the suprachiasmatic nucleus, where long daylight induces short pineal melatonin secretion and increased PT-TSH levels.

The regulation of PT-TSH secretion shows species-specific differences (Figure 2). In seasonally breeding birds, light directly activates PT-TSH secretion [8]. The situation is different in mammals, where PT-TSH secretion is regulated by the pineal hormone melatonin through melatonin receptor 1A (MT1a), not by light. Long days in birds and short melatonin secretion by the pineal gland in mammals increase PT-TSH secretion. The resulting increased expression of DiO2 converts more T4 to T3. Short day length and long melatonin secretion decrease PT-TSH levels leading to relatively higher DiO3 activity, which generates the metabolically inactive reverse T3. The PT thyrotrophs of rats and mice possess melatonin receptors, and accordingly, PT-TSH levels in rats were shown to vary depending on the length of day. Mice, however, are poor models for the study of PT-TSH because the majority of mouse strains (BALB/c, DBA/2, 129/Sv, AKR/J, CF1, and C57Bl/6) have undetectable melatonin levels and the physiological relevance of external melatonin stimulation is questionable [9]. Thyrotrophs of the human PT have low or no MT1a expression and, in contrast to seasonally breeding rhesus monkeys, no binding of melatonin to human PT thyrotrophs has been observed, suggesting that, in humans, melatonin plays no role in the secretion of PT-TSH [10,11].

Recently, a study of over 7000 Japanese subjects impressively demonstrated that TSH in humans is subject to a seasonal rhythm [12]. This rhythm is generally thought to occur via melatonin-mediated circannual fluctuations in TSH secretion, with serum TSH concentrations reaching their annual peak in the northern hemisphere during the winter months (January–February). Through various mechanisms, e.g., meteorological factors, peripheral thyroid hormone concentrations of T3, free triiodothyronine (fT3), and fT4 also adjust to these seasonal fluctuations. However, as shown by Kuzmenko et al., measured T4 concentrations do not exhibit pronounced seasonal dynamics [13]. T4 is the only one of these measured thyroid hormones that is secreted exclusively by the thyroid gland. This suggests that at least part of the serum TSH increase in winter is due to PT-TSH. This TSH isoform has no effect on the thyroid and would explain the unchanged serum T4 levels. Quite analogous to the seasonal serum changes in TSH, changes at the transition between subclinical hypothyroid and euthyroid status are also described [14]. In a big data approach evaluating over 1.5 million data, mean serum TSH levels of adults showed a semi-annual seasonal distribution pattern with higher values in summer and winter regardless of age, sex, and ambient temperatures. Neither fT3 nor fT4 showed a seasonal trend [15]. All in all, the seasonal fluctuations of TSH and their meaning for humans are far from being fully elucidated yet. Further studies are needed. Specific studies should also focus on PT-TSH.

The targets of PT-TSH are the lining cells of the third ventricle of the brain, which include ependymocytes characterized by cilia for the movement of the CSF and tanycytes characterized by the almost complete absence of cilia. Tanycytes are bipolar ependymal cells with long processes that extend into the hypothalamic parenchyma that may link the cerebrospinal fluid (CSF) to neuroendocrine events. They are in close contact with neurons and reach the fenestrated capillary network of the ME, while their tight junctions prevent the diffusion of blood-born molecules into the CSF [16]. They are classified into α and β tanycytes; the existence of γ tanycytes is also hypothesized [17]. β Tanycytes, which have the highest expression of DiO2 of all tanycytes, are located at the base of the third ventricle, while α1 and α2 tanycytes line the lateral walls [18]. The β1 tanycytes form the barrier between the CSF and the tissue of the anterior pituitary gland by a honeycomb pattern of zonula occludens 1- and occludin-immunoreactive tight junctions. Their long processes reach the fenestrated capillary network of the ME and form synapse-like contacts [7]. At the ME, TRH axonal varicosities and terminal buttons reach the capillary vessels that lead to the anterior pituitary gland. Tanycytes regulate TRH secretion in the ME via endocannabinoid release, whereas TRH axons regulate tanycytes by glutamate, suggesting the existence of a reciprocal microcircuit between tanycytes and TRH terminals that controls TRH release [19]. Tanycytes also express a TRH-degrading ectoenzyme (previously termed pyroglutamylpeptidase II) to decrease TRH levels. These mechanisms regulate the amount of TRH entering the pituitary portal vessels, to control energy homeostasis and to regulate feedback to the paraventricular neurons that secrete TRH. PT-TSH binds to TSH receptors of β-tanycytes. By binding to these TSH receptors, PT-TSH regulates DiO2 and DiO3 [20]. The local conversion of inactive thyroxine (T4) to active T3 by DiO2 is increased, whereas the inactivation of T4 to reverse T3/T2 via DIO3 is reduced by the action of PT-TSH. As demonstrated in tanycytes isolated from rats, TSH acted via both activation of adenylate cyclase and phosphorylation of ERK1/2. Furthermore, activation of the TSH receptor leads to an increase in DiO2 mRNA expression [21]. The communication of the PT with the tanycytes is referred to as retrograde action, because PT can also communicate with the PD in an anterograde manner, controlling, e.g., prolactin release via different messengers [22].

Follicle stellate cells (FSCs), characterized by their star-like appearance, represent around 10% of the cells of the anterior pituitary gland [23]. They produce no hormones, but they form a network with each other and with endocrine cells. They possess TSH receptors and are suspected to be involved in the fine-tuning of PD-TSH through ultrashort feedback. They are unique in the way that they express S-100 and glial fibrillary acidic protein (GFAP), secrete growth factors (vascular endothelial growth factor, fibroblast growth factor, and IL-6) and have phagocyte and self-renewal capability [24]. This combination led to the assumption that FSCs play a role in the immune system and further act as stem cells contributing to the maintenance of hormonal cells. The immunoreactivity against DiO2 and the T4 transporter monocarboxylate transporter 8 suggests that FSCs can modify thyroid hormone levels [25]. In the ultrashort regulation of thyroid hormone levels it is assumed that T4 is taken up by FSCs and converted to T3 by DiO2. DiO2 expression is induced by binding of TSH. The ultrashort regulation could cause the pulsatile TSH secretion pattern.

Other types of biological rhythms are circadian, ultradian and infradian rhythms [26]. The circadian rhythm lasts 24 h, while ultradian rhythms are observable, measurable, quantifiable physiological patterns shorter than 24 h (such as pulse, thermoregulation, blinking, appetite, arousal and micturition [3]). Infradian rhythms are longer than 24 h and include the seasonal breeding of some animal species. For humans, the menstrual cycle is a typical example. Circadian, ultradian and infradian rhythms are regulated by the SCN [27,28]. This is a paired structure of the anterior hypothalamus connected to intrinsically photosensitive ganglion cells of the retina via the retinohypothalamic tract [3]. These cells are also functional in >50% of totally blind people, indicating their regulation separate from normal vision. Due to the circadian rhythm, variations in the TSH levels of up to 50% (0–4 am versus 7–9 pm) are within the normal range and do not strictly represent a change in the functional state of the gland [29]. In sleep-deprived individuals, TSH morning concentrations are twice as high as in people with normal sleep. This is due to the fact that sleep inhibits TSH secretion [30]. On the other hand, when the depth of sleep at the habitual time is enhanced due to prior sleep deprivation, the nocturnal TSH rise is markedly reduced. Changes in TSH levels could be associated with the wave pattern of the sleep electroencephalogram, particularly to delta waves activity as indicator for deep sleep. According to a study by Gronfier et al., the nocturnal secretion profile for prolactin and GH correlates with delta wave activity, whereas the nocturnal secretion profile for TSH and cortisol correlate negatively with delta wave activity. It is not clear whether activity shown in electroencephalograms (EEG) has a modulatory role on TSH levels, or inversely, whether TSH variations could influence sleep structure. Alterations in the circadian release pattern of TSH may contribute to thyroid diseases because autoimmune thyroid disorders are more common in shift workers. Night workers with subclinical hypothyroidism (SCH) had higher TSH concentrations than day workers [31]. SCH is linked to overall and subjective sleep quality, shorter sleep latency and a lower proportion of late sleep on weekdays [32]. Action of sleep on TSH secretion may be a reason for this because shorter and longer sleep are linked to SCH [33]. A dysfunction of the hypothalamus pituitary thyroid axis (HPT) can also lead to decreased sleep quality. Staying up late on weekends affects the value of the thyroid homeostasis parameter “structure parameter inference approach-Gain of Thyroid” (SPINA-GT) [32]. This parameter provides an estimate of the maximum thyroid secretory rate under stimulation conditions. It is calculated on the basis of a formula containing the measured TSH and fT4 concentrations and several constants. In addition to PD-TSH, PT-TSH is also linked to sleep quality (see Section 6 below).

Ultradian changes in TSH levels are pulses with a frequency of 20.0 ± 2 min from maximum to maximum [34]. The amount of released TSH at each pulse has been assessed as 0.90 ± 0.06 mIU/L. These changes were not different in males and females but may be age-dependent, although some studies failed to observe any association with age, whereas others found diminished nocturnal surges and their earlier onset in older adults. TRH infusions in human volunteers suggest that TRH may modulate TSH pulse amplitude but, at least acutely, does not determine pulsatile TSH release [35]. One candidate for regulation of TSH levels, independent from TRH and thyroid hormones, may be thyrostimulin, also termed corticotroph-derived glycoprotein hormone (CGH). The protein, as the name suggests, is produced by corticotrophs of the anterior pituitary gland. By binding to the TSH receptor, it increases T4 levels [36]. Its affinity to the TSH receptor is greater than that of TSH and bioactivity is 30 times higher [37]. The stronger affinity is caused by the binding of CGH to both the transmembrane region and the ectodomain of the TSH receptor. It is not clear whether the protein exists in circulating blood because the subunits heterodimerize only at high concentrations. The main function of the protein is seen in the regulation of the pituitary gland to maintain euthyroidism. The authors regarded the pulsative secretion pattern as particularly suitable for responding to rapid changes in the environment.

## 3. Routine TSH Determination in the Laboratory and Interference with the Assays Used

**Figure 3:**
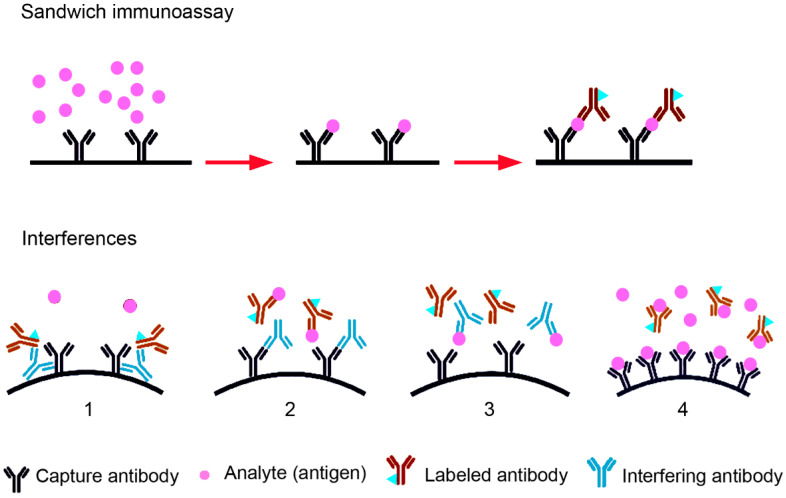
In the sandwich immunoassay, the sample is added to the capture antibody (Ab) and the binding is detected by addition of the labelled Ab. Interferences may occur due to Abs that bridge the capture and the labeled Ab (1), by Abs against Fab fragments of the capture Ab (2), by Ab against the analyte (3), and by excess concentrations of analytes, which saturate all binding sites of capture and labeled Ab (Hook effect, 4).

The determination of TSH, T3 and T4 concentrations in blood is an essential part of routine blood analysis for the assessment of thyroid function. The concentrations are routinely determined by immunoassays. For the detection of small molecules (steroids, T3, T4, vitamin D, etc.), the competitive immunoassay is usually preferred, while larger molecules (e.g., pituitary hormones, calcitonin, thyroglobulin, insulin, inhibin, etc.) are more often detected with a two-side (sandwich) immunoassay [38] (Figure 3). Sandwich assays are usually more robust since antibodies are added in excess and they have higher specificity and lower limits of detection. TSH levels are the critical parameter for the diagnosis of subclinical hypo- or hyperthyroidism. The sensitivity of TSH detection has improved over the years and the limit of detection has decreased in third generation assays from 1 mIU/L to 0.01 mIU/L [39].

Detection with the sandwich immunoassay, routinely used for TSH detection, may be incorrect due to the presence of heterophilic (endogenous) antibodies (Abs) and biotin, and the hook effect. The term “heterophilic antibodies” is generally used for endogenous antibodies of IgG, IgM or IgA isotype binding to the Fc region of assay Abs. Heterophilic Abs are human, naturally occurring, weakly binding, poly-specific antibodies. Heterophilic antibodies have a prevalence of 0.17–40% in the general population [40]. Human anti-animal Abs, by contrast, are monospecific, high-affinity binding Abs, which occur after exposure to animals or animal products, viral and bacterial infections or as autoimmune reaction. Human anti-mouse Abs (HAMAs) are the most common anti-animal Abs. Heterophilic Abs can bridge the capture and the labeled detection Ab in sandwich immunoassays and lead to falsely increased antigen level readouts. The hook effect is caused by excess antigen (analyte) concentrations, which saturate nearly all binding sites of capture and labeled antibody and thus prevent the binding of the detection antibody to the antigen bound to the capture antibody [38]. Apparent antigen concentrations are falsely low because the detection antibody is washed off. Biotin in the sample is a problem when streptavidin-biotin binding is used for separation of the immunocomplex. The presence of biotin in blood samples is not rare because of recommendations to the public to take biotin supplements to improve health or quality of hair and nails. Antibodies can also be directed against the signaling molecules, e.g., against streptavidin, biotin or ruthenium [41,42]. Anti-ruthenium antibodies were shown to mimic macro-TSH in immunoassays using electroluminescent detection [43]. Anti-ruthenium antibodies might be induced by the intake of ruthenium in the food chain or exposure to residues on clothing [44]. The incorrect measurement of TSH concentrations in the presence of macro-TSH is caused by the fact that circulating antibody-TSH complexes produce an abnormal reaction in the immunoassay. The large amounts of TSH are complexed with anti-TSH antibodies but are also detected by the antibodies in the immunoassays. It is debatable if this could be termed “assay interference” because the TSH molecules are present in the blood. Nevertheless, the readout does not reflect the physiological situation because macro-TSH has no biological activity outside of the brain.

TSH concentrations below or above the reference level will result in therapeutic intervention and it is, therefore, important to identify false-positive or false-negative effects. In this context, the existence of macro-TSH has to be taken into consideration. Macro-hormones have been identified for several hypophyseal hormones, namely, adrenocorticotropic hormone (ACTH), follicle-stimulating hormone (FSH) and luteinizing hormone (LH). Prolactin (PRL) is the most frequently detected macro-hormone. PRL exists as monomeric PRL (23 kDa), the dimer big PRL (50–60 kDa) and macro-PRL, also called big-big PRL [45]. Macro-PRL consists of big PRL dimers joined by IgG molecules. It has a molecular weight of 106 Da and no biological activity. Macro-PRL has been found in 10–25% of patients with hyperprolactinemia but only in 4% of the general population. Macro-FSH and macro-LH are generated by the binding of auto-antibodies against no defined epitopes. In the case of macro-PRL the protein region involved in binding to the PRL receptor is suspected to be the main epitope [46,47]. Macro-ACTH results from the incomplete cleavage of pro-opiomelancortin, the prohormone for the generation of ACTH, β-lipoprotein, β-endorphin and α-melanocyte-stimulating hormone (MSH). The immunoreactive but bioinactive ACTH molecules complex with immunoglobulins [48]. As illustrated in the next section, glycosylation of the TSH molecule appears to be an important factor for the formation of macro-TSH.

## 4. The Role of Glycosylation in Macro-TSH Formation

**Figure 4:**
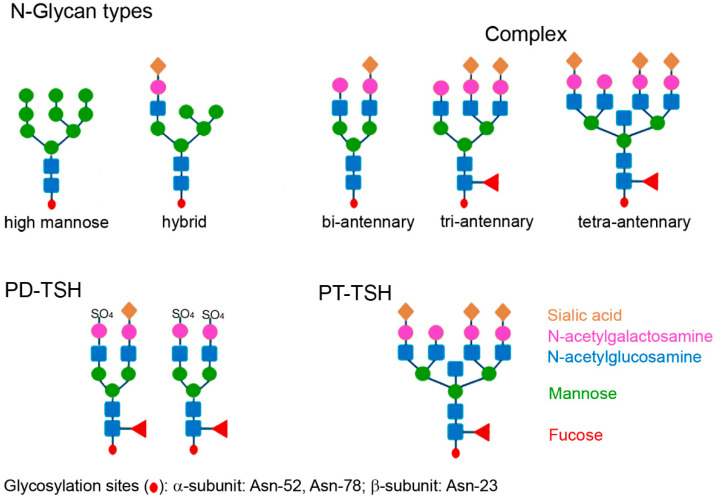
General types of N-Glycosylation in glycoproteins and differences in glycosylation of pars distalis TSH (PD-TSH) and pars tuberalis TSH (PT-TSH).

Glycosylation plays an important role in the biological action of proteins. O-glycosylation, O-GlcNAcylation (O-linked β-N-acetylglucosamine) and N-glycosylation are the main types of glycosylation. The oligosaccharides are attached to the proteins in a multistep enzymatic process occurring in the rough endoplasmic reticulum and the Golgi apparatus, catalyzed by glycosyltransferases. There are, however, differences in the way of synthesis [49]. O-glycosylated glycoproteins are modified post-translationally with oligosaccharide chains synthesized on the protein, by membrane-bound enzymes. Their synthesis is not inhibited by tunicamycin. N-glycosylation is performed co-translationally, where oligosaccharides are transferred en bloc, and enzymes are not membrane-bound and inhibited by tunicamycin. Post-translational N-glycosylation is also possible and mediated by another oligosaccharyltransferase isoform [50]. N-glycosylation is mainly found in eukaryotes, whereas O-glycosylation can occur in both prokaryotes and eukaryotes. N-oligosaccharides are attached via an N-glycosidic bond to asparagine and all N-glycans share the same core structure. The addition of monosaccharides to the core structure leads to the extension of the antennas in the outer part, which may be high mannose (oligomannose), hybrid type or complex type [51]. Complex N-glycosylation is further subclassified into bi-, tri- and tetra-antennary type (Figure 4). The glycosylation of TSH belongs to the complex type. PD-TSH is glycosylated with bi-antennary sulfonated glycans whereas PT-TSH contains sialylated multi-branched glycans. Sialylated glycosylation leads to an increased binding of heterophilic antibodies. The resulting complex of antibodies, albumin and PT-TSH, forming a large molecular-sized TSH, is designated macro-TSH. Macro-hormones are present only in the blood, where heterophile antibodies circulate, but are not present in cell culture medium or in the pituitary gland tissue [52]. This demonstrates that macro-TSH is formed only after contact with immunoglobulins in the blood and that the complex is unable to exit the vascular system to reach receptors on peripheral tissues, such as the thyroid gland. Due to their lack of biological activity, macro-hormones do not contribute to feedback regulation.

TSH is a heterodimer and consists of two subunits, the α and the β subunit. The α subunit is common to other glycoproteins, such as LH and FSH, and human chorionic gonadotropin (hCG), and is responsible for activity, whereas the β subunit is hormone-receptor-specific. There are three potential glycosylation sites in human TSH: two in the α subunit (Asn-52 and Asn-78) and one in the β subunit (Asn-23) [51]. The β subunit may be glycosylated or not glycosylated. Thus, in the bloodstream of all individuals, a triglycosylated molecule and a diglycosylated TSH molecule circulate, but the ratio between them differs [53]. The type of glycosylation usually differs between the subunits. The α subunit contains mainly sialylated and monosulfonated and the β subunit more disulfonated and core-fucosylated structure. The type of glycosylation of the α subunit determines the biological action of TSH. N-glycosylation plays a key role in TSH receptor activation. The glycosylation of the β subunit is important for stability and secretion of the TSH molecule and sialylation of the β subunit decreases metabolic clearance. This is due to the fact that sialic acid hides the N-acetylgalactosamine in the oligosaccharides from the hepatic asialoglycoprotein (galactose-) receptor 1 responsible for clearance of the molecule. The receptor, which is expressed exclusively in the liver, recognizes the GalNAc sulfate signal in desialylated proteins, e.g., PD-TSH, and removes them [54].

In addition to the pulsatile secretion pattern, the glycosylation of TSH is a possibility for regulating thyroid hormone levels. It is assumed that TRH is involved in the regulation of sulfonation and sialylation of TSH [55]. TSH isolated from human pituitary glands typically is a heterogeneous mixture of predominantly GalNAc-sulfated biantennary glycoforms [56]. The molecules have a pH from 6.8 to 8.3 and different bioactivity. Circulating TSH is composed of sialylated glycans. The sulfonated TSH has increased activity for c-AMP production, whereas core fucosylated forms activate the inositol trisphosphate (IP3) pathway more potently. As a general rule, decreased sialylation increases bioactivity and decreases half-life, whereas highly sialylated TSH has prolonged half-life and low intrinsic activity (low affinity to the TSH receptor). Bioactivity is also determined by the number of glycosylation sites (tri- or di-glycosylation).

Primary hypothyroidism, thyroid hormone resistance and TRH administration increase terminal galactosylation and sialylation, resulting in the increased bioactivity of TSH [57]. In children up to 18 months of age, most TSH molecules are high-sulfonated and low-sialylated compared with older children and adults [58]. The low sialylation is similar to TSH of third-trimester fetuses and favors a high bioactivity at thyroid and extrathyroidal TSH receptors. Similarly, pituitary tumors (TSHomas) lead to the production of TSH with enhanced bioactivity [59]. In severe primary hypothyroidism, another adaptation has been observed: the PD-TSH had a lower degree of N-glycosylation, higher degree of sialylation and lower degree of sulfonation than TSH from euthyroid individuals [53]. The TSH molecules of these patients are similar to PT-TSH and display impaired bioactivity due to the high sialylation but longer action due to increased half-life. Inactivating mutations in TRH and TSH receptor lead to mildly increased TSH and diminished T4 concentrations [60]. The bioactivity of TSH in these patients is decreased by increased sialylation of the glycans. Similar to PT-TSH, the increased sialylation is associated with decreased elimination of the molecule [61]. An increase in sialylation is also observed in nighttime compared to daytime TSH [62].

Since TSH immunoreactivity, independent from the glycosylation pattern, is detected by the conventional immunoassays, the ratio of bioactivity to immunoreactivity (B/I ratio) in TSH of patients with primary hypothyroidism and also transiently during nighttime is decreased but can be increased by substitution with L-T4. The decreased bioactivity is observed in vivo, whereas bioactivity in vitro is variable and may also be normal [63]. There is the problem of determining the contributions of the effects on TSH receptor and on renal clearance on the overall effect of TSH with different glycosylation patterns.

PT-TSH is characterized by tri- and tetra-antennary glycans linked with sialic acid. The differences in the glycosylation pattern between TSH produced in the different parts of the pituitary gland are due to the specific expression of glycosyltransferases in the respective TSH-producing cells [64] and influences the binding of IgG and albumin for macro-TSH formation [20]. Albumin has a negative net charge (−15 for humans) at the physiological pH of 7.4 and is the main transport protein in the blood for a variety of endogenous molecules and xenobiotics [65]. Hydrophobic compounds, metabolites, hormones, and nutrients are bound to a greater extent to albumin than (especially negatively) charged molecules. PD-TSH is thought to be more strongly negatively charged due to the sulfate groups, whereas in PT-TSH, the less negative charge of the sialic acid groups may result in a higher binding to albumin. Multi-branched sialylated glycans in PT-TSH also cause increased binding of antibodies. This resulting macro-TSH contains natural antibodies, which may be of IgM or IgG isotype, mainly IgG2b and auto-TSH antibodies [1]. One case of IgA-bound TSH has also been reported [66]. Anti-TSH antibodies are found in patients who received bovine TSH and in patients with autoimmune diseases [67]. The antibodies in patients with macro-TSH were identified as anti-TSH-β auto-antibodies and auto-antibodies against subunit α, a common subunit shared between human TSH, FSH, and LH [68]. The generation of these antibodies is hypothesized to be due to age, reduced autoimmune tolerance and altered TSH- antigenicity [68]. A change in the immunogenic potential may be suspected because macro-TSH contains mainly IgG2. IgG2 is the second most abundant subclass of IgG molecules with 32% compared to IgG1 with 60%, IgG3 with 4% and IgG4 with 4% prevalence [69]. While the other IgG subclasses respond to a greater extent to proteins and allergens, IgG2 is the main responder to polysaccharides. The higher glycosylation of PT-TSH may, therefore, preferentially induce auto-antibodies of this subclass. Macro-TSH is cleared to a lower extent by the kidney, which results in longer circulation times and potential accumulation in the body [70]. This TSH complex is detected in the blood over long periods of time. It lowers the B/I ratio in central hypothyroidism even more than sialylated TSH.

## 5. Identification and Prevalence of Macro-TSH

The presence of macro-TSH can result in an under-estimation of TSH if the TSH-epitopes recognized by the immunoassay antibody are occupied by the complex of PT-TSH with endogenous antibodies [71]. There may also be an overestimation of TSH concentrations when the antibodies cross-link the capture and the labelled antibody of the immunoassay. Several techniques help in the identification of macro-TSH. Macro-hormones are often heterogeneous, and immunoassays produce different results. Therefore, the use of more than one immunoassay may be necessary to identify macro-TSH [72]. The Abbott Architect TSH assay was found to be less affected by macro-TSH in the sample than Roche E170 Modular Analytics and Advia Centaur assays in that study. According to another study, the E170 Modular Analytics (Roche Diagnostics) was found to be more sensitive than the Advia Centaur from Siemens Healthcare Diagnostics [73]. Rix et al. found that E170 Modular Analytics and Perkin-Elmer Delfia assays are very sensitive for macro-TSH and Advia Centaur less sensitive [74]. In the comparison between E170 Modular Analytics assay, Beckman Coulter Access TSH and Advia Centaur, the latter was less sensitive for macro-TSH than the former two assays [75].

The incubation of the sample with serum from hypothyroid patients (1 + 1) is another method to reveal the existence of macro-TSH [8]. When macro-TSH is present in the sample, it will markedly reduce the TSH levels of the hypothyroid serum because some of the native TSH has been converted to the less detectable macro-TSH. However, the reduction in TSH recovery occurs to a lesser extent in TSH assays that are sensitive to macro-TSH because these assays can still detect considerable amounts of the newly formed macro-TSH. Hence, assays that are less sensitive to macro-TSH are better suited for this investigation because the fall in TSH recovery will be more pronounced. The presence of macro-TSH is also suspected when the signal of diluted samples lacks linearity or when specific measures (use of heterophile blocking tubes or protein A pretreatment) markedly lower the content of TSH in the sample [38].

A decrease in TSH after immunocomplex precipitation with polyethylene glycol (PEG) by >75% of the untreated sample also indicates the presence of macro-hormones. PEG competes with the immunoglobulin surface for water molecules. When less water is available to hydrate the surface, then immunoglobulin molecules start to precipitate. With this method IgG and IgM can be fairly specifically precipitated. By contrast, only 50% of IgA is precipitable. Upon addition of the IgA-binding lectin Jacalin, IgA also can be adequately precipitated [66]. In one study, TSH concentrations decreased after precipitation with PEG in 30–40% of patients without thyroid disease or thyroid cancer and in 63% of patients with SCH according to another study [76]. The precipitation method as an indication for the presence of macro-TSH is somewhat controversial because increased concentrations of globulins in the blood may also augment the fraction of precipitated TSH [39]. In a study by Giusti et al., PEG-precipitable TSH was 39.3% ± 1.9% in thyroid cancer patients and 44.1% ± 3.9% in controls [77]. After PEG precipitation and applying the cut-off levels of >80%, only 3.1% of samples from thyroid cancer patients contained macro-TSH. This prevalence was higher than values reported for SCH from other groups, e.g., 0.79–1.6% [72,76]. Due to this relatively high background precipitation rate, macro-TSH is only suspected when the signal decreases by more than 75% of the initial value. Protein A and protein G precipitate almost all Ig classes. Ohba et al. suggested the use of cut-off levels based on the relation of TSH and free thyroxine (fT4) as Log10TSH = 0.700 + 1.549/(1 + (fT4/0.844)) [78]. With this method, the authors could identify macro-TSH in 80% of the positive samples.

Gel filtration chromatography (GFC) is the gold standard for the verification of macro-TSH in the sample but is more expensive and less accessible than the other techniques. This method may also be prone to errors because it can confound macro-TSH with HAMAs, which elute from the column at the same position as γ-globulin. This implies that screening for HAMAs must be performed in parallel.

Manufacturers of immunoassays developed strategies, such as the inclusion of non-immune animal Ig from the same species as a scavenger for antibodies, aggregated Ig, chimerized or humanized antibodies, and Fab fragments or sheep antibodies, to overcome the problem of interference with HAMAs [38]. The suggested workflow for the identification of macro-TSH is precipitation with PEG in samples with TSH concentrations > 10 mU/L followed by confirmation with gel chromatography [67].

Many reports on macro-TSH are case studies [79,80,81,82,83,84], suggesting that the existence of macro-TSH is not generally known. Macro-TSH was reported with a prevalence of 0.6% in individuals with TSH concentrations > 10 mIU/L [75]. In patients with SCH, 0.79–1.6% of the samples contained macro-TSH [72,76]. Other studies report prevalence in a similar range in these patients: 1.6% as verified with gel filtration chromatography (GFC) and 0.8% upon the exclusion of HAMA [76]. In patients with thyroid cancer, 3.1% of the samples contained macro-TSH [77]. The prevalence was higher in older adults and in females. A much higher prevalence of macro-TSH was reported in a Russian study [85]. The authors detected macro-TSH in 53.3% of patients with SCH and in 25% of healthy controls. Immunoglobulin bound to TSH was identified via PEG precipitation, GFC and protein A binding. The authors explained the high prevalence of macro-TSH by the fact that they studied patients with >10 ImU/L concentrations. However, the percentage in their healthy controls was also considerable higher than the prevalence reported in other studies. Hattori et al. re-evaluated a small patient collective and found that macro-TSH persisted in 11/13 patients over a one- to four-year period after the first evaluation. Serum TSH levels returned to normal in the remaining two patients whose macro-TSH disappeared [68]. Although high macro-TSH levels are seen in some patients with SCH and overt hypothyroidism, its presence is not linked to thyroid dysfunction but is also found in individuals with normal TSH levels [86].

To better identify patients with increased TSH levels due to macro-TSH, Favresse et al. suggested to screen patients with isolated TSH elevation (typically markedly elevated), in whom thyroid hormones are in the upper half of the normal range, and who have no signs or symptoms in thyroid dysfunction [39]. It seems possible that the same patient may have falsely elevated TSH concentrations due to the simultaneous occurrence of heterophilic antibodies and an erroneous laboratory result due to a biotin interference. In addition, a fraudulently elevated FT4 concentration due to thyroid hormone autoantibodies may also be present [87].

The identification of elevated TSH concentrations is difficult due to differences in the values between the immunoassays and problems in the setting of normal ranges (reference intervals, RIs). Aliquots of a serum sample in which TSH is measured should give identical results independent of laboratory equipment, personnel, and environment and assay method [88]. This can be achieved by standardization or by harmonization. Standardization is a process by which comparable results are obtained by having each laboratory’s methods of calibration traceable to a reference measurement procedure (RMP). The variable glycosylation pattern and TSH-unrelated specificity problems prevent the development of a RMP and TSH assays are therefore harmonized. Harmonization describes the use of a reference system consisting of methods and materials that are not traceable to the système international d’unités (SI) but are agreed upon to act as references. The National Academy of Clinical Biochemistry (NACB) suggested selecting at least 120 individuals with the following inclusion criteria: (i) no personal or family history of thyroid disease, (ii) negative for thyroid antibodies, (iii) presenting no visible or palpable goiter, and (iv) not receiving any treatments affecting thyroid function (except estrogen). The 2.5th and 97.5th percentiles of the log-transformed TSH are identified as the RI for TSH [89]. This number of individuals may be too small because outliers strongly affect the upper limit. In a multicenter study, recalibration equations were derived based on three immunoassays, the Access 3rd IS Thyrotropin (Beckman Coulter Diagnostics), the Architect system (Abbott Diagnostics) and the Elecsys (Roche Diagnostics) [90]. After validation using an external quality assessment, it was observed that recalibration decreased the coefficients of variation from 10.72% to 8.16%. Further, method-independent RIs for TSH were obtained.

**Figure 5:**
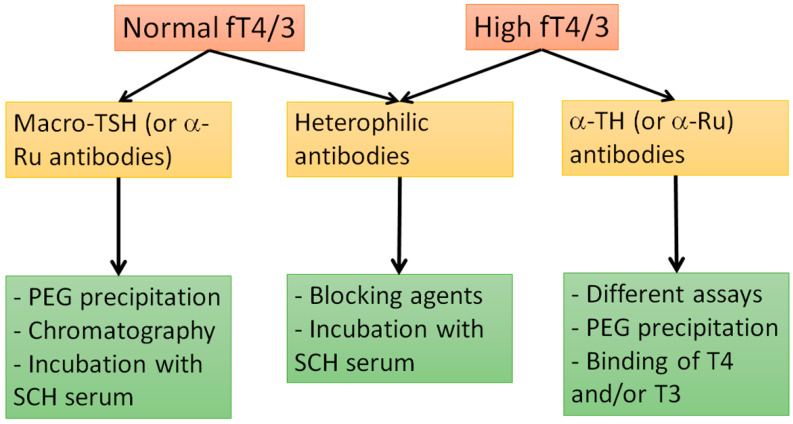
Algorithm for the identification of macro-TSH (content according to [61]). Findings are written in red, reasons in orange and measures in green boxes. Ruthenium (Ru) was used for automated detection of TSH in the Elecsys^®^ TSH assay (Roche Diagnostics; https://labogids.sintmaria.be/sites/default/files/files/tsh_2018-05_v24.pdf (accessed on 14 May 2023)). Abbreviation: α-Ru, anti-ruthenium; α-TH; anti-thyroid hormone; SCH, subclinical hypothyroidism.

When there is a discrepancy between thyroid laboratory parameters and clinical manifestation, the presence of immunoassay interference with one or more indicators (T4, TSH) needs to be considered. In this case, the following measures shown in Figure 5 should be taken.

## 6. Characteristics of Macro-TSH and Hypotheses concerning Its Role in Humans

In a young male with macro-thyrotropinemia, it was observed that short-term L-T4 substitution significantly decreased plasma TSH to near-normal TSH levels within 2–4 weeks, but hyperthyroid symptoms emerged [71]. The authors suggested that when the secretion of freely circulating TSH from the PD is suppressed, TSH could be released from the antibody complex. In this case, the situation would be similar to free and bound thyroid hormones, where only a minor fraction is freely circulating. This theory was based on the fact that, if the binding in the TSH complex was irreversible, the decline in TSH would have been more prolonged, because the half-life of IgG is about 30 days [91]. But this theory is made less likely by the finding that TSH-Ig complexes were very stable and dissociated only at pH 3.0 [92]. According to another hypothesis, the complex formation of TSH with immunoglobulin or albumin serves to remove damaged protein similar to protein binding for the inactivation of drugs [1]. Such a function cannot be excluded, although the elimination of the complexed TSH is much slower than that of the free TSH. In another case study, macro-TSH prevented the increase in fT3 in the TRH stimulation test despite a normal PRL response. The authors proposed a rapid binding of PD-TSH, secreted after TRH stimulation, to heterophilic antibodies forming the hormonally inactive macro-TSH, which therefore did not increase serum fT3, but did not provide a proof for the hypothesized mechanism [78].

The absent/low bioactivity and the lack of association of macro-thyrotropinemia with pathologies or concrete symptoms suggests that macro-TSH has no positive or negative effects in humans. Macro-PRL, which is more common than macro-TSH, was reported to have neither a specific physiological nor a pathological role [93]. Increased TSH levels in patients with hyperprolactinemia suggest a common tendency for the formation of anti-hormone antibodies [94]. Since 72.3% of patients with hyperprolactinemia had TSH levels in the upper range (>4.2 mIU/L), it may be hypothesized that macro-TSH was present in the samples due to the cross-reactivity of anti-PRL antibodies [94]. There was also a high frequency of patients with hyperprolactinemia (21% and 46%) with autoimmune thyroid disorders [95,96]. The reverse correlation, hyperprolactinemia in patients with SCH, was reported, with a prevalence of 8–34.93% [94,97].

In a study including patients with cardiovascular risk factors, according to PEG precipitation, in 50% of patients, macro-TSH represented >80% of total TSH [98]. These patients also had higher body mass indices, and a higher prevalence of dyslipidemia and diabetes mellitus than the group with lower macro-TSH levels. After adjustment for these parameters, a significant correlation of macro-TSH with sleep physical activity and percent sleep was found. They found that serum macro-TSH was significantly correlated with fasting glucose, HbA1c, and the homeostasis model assessment of insulin resistance (HOMA-IR), a simple and useful method for evaluating insulin sensitivity. Macro-TSH levels were much higher in type 2 diabetes mellitus (T2DM) patients than in the non-diabetic controls. T2DM may result in an increased and/or altered glycosylation of TSH [99], facilitating the formation of macro-TSH. It is further possible that the increased IgG2 levels, which were found in T2DM patients with high body mass index, waist circumference and complement C3 levels, lead to increased complex formation with circulating TSH [100]. This IgG class is relevant because it is mainly contained in macro-TSH [1]. The increased levels of macro-PRL in T2DM patients compared to normal controls may suggest a general mechanism in these patients leading to macro-hormone formation [101].

From the diagnostic point of view, knowledge of the presence of macro-TSH in the sample is important because increased TSH levels have therapeutic implications. Failure to identify macro-TSH in the blood of neonates may lead to the diagnosis of hypothyroidism and to T4 replacement therapy [8]. Macro-TSH was detected in 0.43% of neonates and also in their mothers [102]. It is hypothesized that in pregnant women, maternal anti-TSH antibodies cross the placental barrier and form macro-TSH complexes with neonatal TSH. Therefore, it would be good to also screen the mother for TSH and fT4 to avoid a pitfall. If the mother also presents elevated TSH levels and normal fT4, macro-TSH should be considered as a possible reason. In this situation, the neonate should not be treated with L-T4 but the screening should be repeated after several weeks because the macro-TSH transferred from the mother will have disappeared by then. Also in adults, a lack of identification of macro-TSH may result in therapeutic problems. One case study reported inadequate treatment of a patient with Graves’ disease with L-T4 based on falsely increased TSH concentrations caused by the presence of macro-TSH [103].

Interference by macro-TSH should be further differentiated from the inappropriate secretion of TSH (IST), which is characterized by increased TSH and increased fT4 and fT3 concentrations [104]. This situation is sometimes called central hyperthyroidism. The causes of IST may be the neoplastic production of TSH (TSHoma in the pituitary gland or ectopic TSHoma) or the non-neoplastic pituitary hypersecretion of TSH (resistance to thyroid hormone beta, TRH administration, and Cushing’s syndrome after surgical resection). The term syndrome of IST (SITSH) is used when only fT4 and not fT3 is increased. Unlike IST, elevated fT3 is not a criterion for SITSH.

## 7. Conclusions

Glycosylation patterns define the bioactivity and macro-TSH formation of PD-TSH and PT-TSH. While PT-TSH has central but no peripheral activity and has a greater propensity to form macro-TSH, PD-TSH is the main stimulator of the thyroid gland. In contrast to other species, human PT-TSH appears to play only a minor role in biological rhythms. It is likely that macro-TSH itself has no physiological or pathological role in humans. The identification of macro-TSH in the sample is nevertheless important to avoid unnecessary supplementation with L-T4 inducing iatrogenic hyperthyroidism, particularly in neonates. To solve this problem as simply as possible in the future, TSH determination methods that do not interfere with macro-TSH will be required.

## Data Availability

No new data were created or analyzed in this study. Data sharing is not applicable to this article.

## Abbreviations

Abs	Antibodies
ACTH	Adrenocorticotropic hormone
BBB	Blood–brain barrier
CGH	Corticotroph-derived glycoprotein hormone
CSF	Cerebrospinal fluid
DiO2	Deiodinase type 2
DiO3	Deiodinase type 3
EEG	Electroencephalogram
FSC	Follicle stellate cell
FSH	Follicle stimulating hormone
fT3, fT4	Free triiodothyronine, free thyroxine
GFAP	Glial fibrillary acidic protein
GFC	Gel filtration chromatography
HAMA	Human anti-mouse Antibodies
hCG	Human chorionic gonadotropin
HOMA-IR	Homeostasis model assessment of insulin resistance
HPT	Hypothalamus pituitary thyroid
IP3	Inositol trisphosphate
IST	Inappropriate secretion of TSH
LH	Luteinizing hormone
MT1a	Melatonin receptor 1a
ME	Median eminence
MSH	Melanocyte-stimulating hormone
NACB	National academy of clinical biochemistry
PD	Pars distalis
PEG	Polyethylene glycol
PFA	Perifornical area
RMP	Reference measurement procedure
RI	Reference interval
PT	Pars tuberalis
PRL	Prolactin
SCH	Subclinical hypothyroidism
SCN	Suprachiasmatic nucleus
SI	Système international d’unités
SITSH	Syndrome of inappropriate secretion of TSH
SPINA-GT	Thyroid homeostasis parameter structure parameter inference approach-Gain of Thyroid
T3	Triiodothyronine
T4	Thyroxine
T2DM	Type 2 diabetes mellitus
TRH	Thyrotropin releasing hormone
TSH	Thyroid-stimulating hormone
V	Ventricle

## References

[1] Ikegami K., Liao X.H., Hoshino Y., Ono H., Ota W., Ito Y., Nishiwaki-Ohkawa T., Sato C., Kitajima K., Iigo M. (2014). Tissue-specific posttranslational modification allows functional targeting of thyrotropin. Cell Rep..

[2] Kalsbeek A., Fliers E., Franke A.N., Wortel J., Buijs R.M. (2000). Functional connections between the suprachiasmatic nucleus and the thyroid gland as revealed by lesioning and viral tracing techniques in the rat. Endocrinology.

[3] Ikegami K., Refetoff S., Van Cauter E., Yoshimura T. (2019). Interconnection between circadian clocks and thyroid function. Nat. Rev. Endocrinol..

[4] Chiamolera M.I., Wondisford F.E. (2009). Thyrotropin-Releasing Hormone and the Thyroid Hormone Feedback Mechanism. Endocrinology.

[5] Hanon E.A., Lincoln G.A., Fustin J.M., Dardente H., Masson-Pévet M., Morgan P.J., Hazlerigg D.G. (2008). Ancestral TSH mechanism signals summer in a photoperiodic mammal. Curr. Biol..

[6] Dash P. (2020). Blood Brain Barrier and Cerebral Metabolism. Homeostasis and Higher Brain Functions.

[7] Korf H.W., Møller M. (2021). Arcuate nucleus, median eminence, and hypophysial pars tuberalis. Handb. Clin. Neurol..

[8] Loh T.P., Kao S.L., Halsall D.J., Toh S.A., Chan E., Ho S.C., Tai E.S., Khoo C.M. (2012). Macro-thyrotropin: A case report and review of literature. J. Clin. Endocrinol. Metab..

[9] Kennaway D., Voultsios A., Varcoe T., Moyer R. (2002). Melatonin in mice: Rhythms, response to light, adrenergic stimulation, and metabolism. Am. J. Physiol. Regul. Integr. Comp. Physiol..

[10] Weaver D.R., Stehle J.H., Stopa E.G., Reppert S.M. (1993). Melatonin receptors in human hypothalamus and pituitary: Implications for circadian and reproductive responses to melatonin. J. Clin. Endocrinol. Metab..

[11] Wu Y.H., Zhou J.N., Balesar R., Unmehopa U., Bao A., Jockers R., Van Heerikhuize J., Swaab D.F. (2006). Distribution of MT1 melatonin receptor immunoreactivity in the human hypothalamus and pituitary gland: Colocalization of MT1 with vasopressin, oxytocin, and corticotropin-releasing hormone. J. Comp. Neurol..

[12] Yamada S., Horiguchi K., Akuzawa M., Sakamaki K., Shimomura Y., Kobayashi I., Andou Y., Yamada M. (2022). Seasonal Variation in Thyroid Function in Over 7,000 Healthy Subjects in an Iodine-sufficient Area and Literature Review. J. Endocr. Soc..

[13] Kuzmenko N.V., Tsyrlin V.A., Pliss M.G., Galagudza M.M. (2021). Seasonal variations in levels of human thyroid-stimulating hormone and thyroid hormones: A meta-analysis. Chronobiol. Int..

[14] Fu J., Zhang G., Xu P., Guo R., Li J., Guan H., Li Y. (2021). Seasonal Changes of Thyroid Function Parameters in Women of Reproductive Age Between 2012 and 2018: A Retrospective, Observational, Single-Center Study. Front. Endocrinol..

[15] Santi D., Spaggiari G., Brigante G., Setti M., Tagliavini S., Trenti T., Simoni M. (2019). Semi-annual seasonal pattern of serum thyrotropin in adults. Sci. Rep..

[16] Contet C., Goulding S.P., Kuljis D.A., Barth A.L., Candice C. (2016). International Review of Neurobiology.

[17] Wittmann G., Farkas E., Szilvásy-Szabó A., Gereben B., Fekete C., Lechan R.M. (2017). Variable proopiomelanocortin expression in tanycytes of the adult rat hypothalamus and pituitary stalk. J. Comp. Neurol..

[18] Rodríguez-Rodríguez A., Lazcano I., Sánchez-Jaramillo E., Uribe R.M., Jaimes-Hoy L., Joseph-Bravo P., Charli J.L. (2019). Tanycytes and the Control of Thyrotropin-Releasing Hormone Flux Into Portal Capillaries. Front. Endocrinol..

[19] Farkas E., Varga E., Kovács B., Szilvásy-Szabó A., Cote-Vélez A., Péterfi Z., Matziari M., Tóth M., Zelena D., Mezriczky Z. (2020). A Glial-Neuronal Circuit in the Median Eminence Regulates Thyrotropin-Releasing Hormone-Release via the Endocannabinoid System. iScience.

[20] Nakayama T., Yoshimura T. (2018). Seasonal Rhythms: The Role of Thyrotropin and Thyroid Hormones. Thyroid.

[21] Bolborea M., Helfer G., Ebling F.J., Barrett P. (2015). Dual signal transduction pathways activated by TSH receptors in rat primary tanycyte cultures. J. Mol. Endocrinol..

[22] Wood S., Loudon A. (2018). The pars tuberalis: The site of the circannual clock in mammals?. Gen. Comp. Endocrinol..

[23] Prummel M.F., Brokken L.J., Wiersinga W.M. (2004). Ultra short-loop feedback control of thyrotropin secretion. Thyroid.

[24] Pires M., Tortosa F. (2006). Update on Pituitary Folliculo-Stellate Cells. Int. Arch. Endocrinol. Clin. Res..

[25] Fliers E., Unmehopa U.A., Alkemade A. (2006). Functional neuroanatomy of thyroid hormone feedback in the human hypothalamus and pituitary gland. Mol. Cell. Endocrinol..

[26] Pfaff D.W., Rubin R.T., Schneider J.E., Head G.A., Pfaff D.W., Rubin R.T., Schneider J.E., Head G.A. (2018). Principles of Hormone/Behavior Relations.

[27] Coomans C.P., Ramkisoensing A., Meijer J.H. (2015). The suprachiasmatic nuclei as a seasonal clock. Front. Neuroendocrinol..

[28] Hu K., Scheer F.A., Ivanov P., Buijs R.M., Shea S.A. (2007). The suprachiasmatic nucleus functions beyond circadian rhythm generation. Neuroscience.

[29] Sheehan M.T. (2016). Biochemical Testing of the Thyroid: TSH is the Best and, Oftentimes, Only Test Needed—A Review for Primary Care. Clin. Med. Res..

[30] Gronfier C., Brandenberger G. (1998). Ultradian rhythms in pituitary and adrenal hormones: Their relations to sleep. Sleep Med. Rev..

[31] Coppeta L., Di Giampaolo L., Rizza S., Balbi O., Baldi S., Pietroiusti A., Magrini A. (2020). Relationship between the night shift work and thyroid disorders: A systematic review and meta-analysis. Endocr. Regul..

[32] Wu K., Zhou Y., Ke S., Huang J., Gao X., Li B., Lin X., Liu X., Liu X., Ma L. (2021). Lifestyle is associated with thyroid function in subclinical hypothyroidism: A cross-sectional study. BMC Endocr. Disord..

[33] Kim W., Lee J., Ha J., Jo K., Lim D.J., Lee J.M., Chang S.A., Kang M.I., Kim M.H. (2019). Association between Sleep Duration and Subclinical Thyroid Dysfunction Based on Nationally Representative Data. J. Clin. Med..

[34] Van der Spoel E., Roelfsema F., Van Heemst D. (2021). Within-Person Variation in Serum Thyrotropin Concentrations: Main Sources, Potential Underlying Biological Mechanisms, and Clinical Implications. Front. Endocrinol..

[35] Samuels M.H., Henry P., Luther M., Ridgway E.C. (1993). Pulsatile TSH secretion during 48-hour continuous TRH infusions. Thyroid.

[36] Okada S.L., Ellsworth J.L., Durnam D.M., Haugen H.S., Holloway J.L., Kelley M.L., Lewis K.E., Ren H., Sheppard P.O., Storey H.M. (2006). A glycoprotein hormone expressed in corticotrophs exhibits unique binding properties on thyroid-stimulating hormone receptor. Mol. Endocrinol..

[37] Karponis D., Ananth S. (2017). The role of thyrostimulin and its potential clinical significance. Endocr. Regul..

[38] Ghazal K., Brabant S., Prie D., Piketty M.L. (2022). Hormone Immunoassay Interference: A 2021 Update. Ann. Lab. Med..

[39] Favresse J., Burlacu M.C., Maiter D., Gruson D. (2018). Interferences With Thyroid Function Immunoassays: Clinical Implications and Detection Algorithm. Endocr. Rev..

[40] Morton A. (2014). When lab tests lie … heterophile antibodies. Aust. Fam. Physician.

[41] Rulander N.J., Cardamone D., Senior M., Snyder P.J., Master S.R. (2013). Interference from anti-streptavidin antibody. Arch. Pathol. Lab. Med..

[42] Vos M.J., Rondeel J.M.M., Mijnhout G.S., Endert E. (2017). Immunoassay interference caused by heterophilic antibodies interacting with biotin. Clin. Chem. Lab. Med..

[43] Gessl A., Blueml S., Bieglmayer C., Marculescu R. (2014). Anti-ruthenium antibodies mimic macro-TSH in electrochemiluminescent immunoassay. Clin. Chem. Lab. Med..

[44] Buijs M.M., Gorgels J.P., Endert E. (2011). Interference by antiruthenium antibodies in the Roche thyroid-stimulating hormone assay. Ann. Clin. Biochem..

[45] Saleem M., Martin H., Coates P. (2018). Prolactin Biology and Laboratory Measurement: An Update on Physiology and Current Analytical Issues. Clin. Biochem. Rev..

[46] De Oliveira Andrade L.J., Matos de Oliveira G.C. (2021). “Incidentalormones”—Macro-hormones. SciELO.

[47] Kasum M., Oreskovic S., Zec I., Jezek D., Tomic V., Gall V., Adzic G. (2012). Macroprolactinemia: New insights in hyperprolactinemia. Biochem. Med..

[48] Day R., Squire L.R. (2009). Encyclopedia of the Neuroscience.

[49] Varki A., Cummings R., Esko J., Stanly P., Hart G., Aebi M., Mohnen D., Kinoshita T., Packer N., Prestegard J. (2022). Essentials of Glycobiology.

[50] Ruiz-Canada C., Kelleher D.J., Gilmore R. (2009). Cotranslational and posttranslational N-glycosylation of polypeptides by distinct mammalian OST isoforms. Cell.

[51] Ząbczyńska M., Kozłowska K., Pocheć E. (2018). Glycosylation in the Thyroid Gland: Vital Aspects of Glycoprotein Function in Thyrocyte Physiology and Thyroid Disorders. Int. J. Mol. Sci..

[52] Fahie-Wilson M. (2003). In hyperprolactinemia, testing for macroprolactin is essential. Clin. Chem..

[53] Wide L., Eriksson K. (2021). Thyrotropin N-glycosylation and Glycan Composition in Severe Primary Hypothyroidism. J. Endocr. Soc..

[54] Donadio S., Pascual A., Thijssen J.H., Ronin C. (2006). Feasibility study of new calibrators for thyroid-stimulating hormone (TSH) immunoprocedures based on remodeling of recombinant TSH to mimic glycoforms circulating in patients with thyroid disorders. Clin. Chem..

[55] Gesundheit N., Magner J.A., Chen T., Weintraub B.D. (1986). Differential sulfation and sialylation of secreted mouse thyrotropin (TSH) subunits: Regulation by TSH-releasing hormone. Endocrinology.

[56] Szkudlinski M.W., Fremont V., Ronin C., Weintraub B.D. (2002). Thyroid-stimulating hormone and thyroid-stimulating hormone receptor structure-function relationships. Physiol. Rev..

[57] Trojan J., Theodoropoulou M., Usadel K.H., Stalla G.K., Schaaf L. (1998). Modulation of human thyrotropin oligosaccharide structures—Enhanced proportion of sialylated and terminally galactosylated serum thyrotropin isoforms in subclinical and overt primary hypothyroidism. J. Endocrinol..

[58] Wide L., Eriksson K. (2019). Unique Pattern of N-Glycosylation, Sialylation, and Sulfonation on TSH Molecules in Serum of Children Up to 18 Months. J. Clin. Endocrinol. Metab..

[59] Beck-Peccoz P., Persani L. (1994). Variable biological activity of thyroid-stimulating hormone. Eur. J. Endocrinol..

[60] Persani L., Ferretti E., Borgato S., Faglia G., Beck-Peccoz P. (2000). Circulating thyrotropin bioactivity in sporadic central hypothyroidism. J. Clin. Endocrinol. Metab..

[61] Estrada J.M., Soldin D., Buckey T.M., Burman K.D., Soldin O.P. (2014). Thyrotropin isoforms: Implications for thyrotropin analysis and clinical practice. Thyroid.

[62] Persani L., Borgato S., Romoli R., Asteria C., Pizzocaro A., Beck-Peccoz P. (1998). Changes in the degree of sialylation of carbohydrate chains modify the biological properties of circulating thyrotropin isoforms in various physiological and pathological states. J. Clin. Endocrinol. Metab..

[63] Horimoto M., Nishikawa M., Ishihara T., Yoshikawa N., Yoshimura M., Inada M. (1995). Bioactivity of thyrotropin (TSH) in patients with central hypothyroidism: Comparison between in vivo 3,5,3′-triiodothyronine response to TSH and in vitro bioactivity of TSH. J. Clin. Endocrinol. Metab..

[64] Ertek S. (2021). Molecular economy of nature with two thyrotropins from different parts of the pituitary: Pars tuberalis thyroid-stimulating hormone and pars distalis thyroid-stimulating hormone. Arch. Med. Sci..

[65] Peters T.J. (1996). All about Albumin: Biochemistry, Genetics, and Medical Applications.

[66] Fukushita M., Watanabe N., Yoshimura Noh J., Yoshihara A., Matsumoto M., Suzuki N., Yoshimura R., Sugino K., Ito K. (2021). A case of macro-TSH consisting of IgA-bound TSH. Endocr. J..

[67] Orgiazzi J. (2021). The Concept of Macro-TSH Revisited. Clin. Thyroidol..

[68] Hattori N., Ishihara T., Matsuoka N., Saito T., Shimatsu A. (2017). Anti-thyrotropin autoantibodies in patients with macro-thyrotropin and long-term changes in macro-thyrotropin and serum thyrotropin levels. Thyroid.

[69] Vidarsson G., Dekkers G., Rispens T. (2014). IgG subclasses and allotypes: From structure to effector functions. Front. Immunol..

[70] Richa V., Rahul G., Sarika A. (2010). Macroprolactin; a frequent cause of misdiagnosed hyperprolactinemia in clinical practice. J. Reprod. Infertil..

[71] Larsen C.B., Petersen E.R.B., Overgaard M., Bonnema S.J. (2021). Macro-TSH: A Diagnostic Challenge. Eur. Thyroid J..

[72] Hattori N., Ishihara T., Shimatsu A. (2016). Variability in the detection of macro TSH in different immunoassay systems. Eur. J. Endocrinol..

[73] Mendoza H., Connacher A., Srivastava R. (2009). Unexplained high thyroid stimulating hormone: A “BIG” problem. BMJ Case Rep..

[74] Rix M., Laurberg P., Porzig C., Kristensen S.R. (2011). Elevated thyroid-stimulating hormone level in a euthyroid neonate caused by macro thyrotropin-IgG complex. Acta Paediatr..

[75] Mills F., Jeffery J., Mackenzie P., Cranfield A., Ayling R.M. (2013). An immunoglobulin G complexed form of thyroid-stimulating hormone (macro thyroid-stimulating hormone) is a cause of elevated serum thyroid-stimulating hormone concentration. Ann. Clin. Biochem..

[76] Picazo-Perea M., Ruiz-Gines M., Ruiz-Gines J., Sastre-Marcos J., Agudo-Macazaga M., Lorenzo-Lozano M. (2021). Macro-TSH in COVID-19 Patients with an Underlying Thyroid Condition: A Case Series and Literature Review. Ann. Thyroid Res..

[77] Giusti M., Conte L., Repetto A.M., Gay S., Marroni P., Mittica M., Mussap M. (2017). Detection of Polyethylene Glycol Thyrotropin (TSH) Precipitable Percentage (Macro-TSH) in Patients with a History of Thyroid Cancer. Endocrinol. Metab..

[78] Ohba K., Maekawa M., Iwahara K., Suzuki Y., Matsushita A., Sasaki S., Oki Y., Nakamura H. (2020). Abnormal thyroid hormone response to TRH in a case of macro-TSH and the cut-off value for screening cases of inappropriate TSH elevation. Endocr. J..

[79] Peynirci H., Ersoy C., Sahin A., Imamoglu S. (2014). Macro-TSH Can be a Rare Cause of Elevated Serum Thyroid Stimulating Hormone Concentration: A Case Report. Med. Sci..

[80] D’Arcy R., Hunter S., Spence K., McDonnell M. (2021). A Case of macro-TSH masquerading as subclinical hypothyroidism. BMJ Case Rep..

[81] Kirac C.O., Abusoglu S., Paydas Hataysal E., Kebapcilar A., Ipekci S.H., Ünlü A., Kebapcilar L. (2020). A rare cause of subclinical hypothyroidism: Macro-thyroid-stimulating hormone. Diagnosis.

[82] Hattori N., Ishihara T., Yamagami K., Shimatsu A. (2015). Macro TSH in patients with subclinical hypothyroidism. Clin. Endocrinol..

[83] Tamaki H., Takeoka K., Nishi I., Shindoh Y., Tsukada Y., Amino N. (1995). Novel thyrotropin (TSH)-TSH antibody complex in a healthy woman and her neonates. Thyroid.

[84] Verhoye E., Van den Bruel A., Delanghe J.R., Debruyne E., Langlois M.R. (2009). Spuriously high thyrotropin values due to anti-thyrotropin antibodies in adult patients. Clin. Chem. Lab. Med..

[85] Biktagirova E.M., Vagapova G.R., Semakov G.P., Zolotoverkchova N.I., Nevzorova T.A., Andrianova I.A., Evtyugina N.G., Akberova N.I., Khisamutdinov A.N., Abramova Z.I. (2019). Detection of macro-thyrotropinaemia in patients with Hashimotos thyroiditis and subclinical hypothyroidism. Med. Immunol..

[86] Lewis E., Lim R., Joseph F., Ewins D., Goenka N., Bowles S., Faye S., Kertesz G. (2011). Recognising macro-TSH: A rare cause of inappropriately high TSH values. Clin. Chem. Lab. Med..

[87] Ni J., Yu L., Li J., Zhang L., Yang Q., Kou C., Li S., Tian G., Wang Y., Liu X. (2021). Interference Due to Heterophilic Antibody, Biotin and Thyroid Hormone Autoantibody. Res. Sq..

[88] The American Thyroid Association (2019). Standardization and Harmonization. https://www.thyroid.org/wp-content/uploads/publications/lab-services/ata-harmonization-standardization.pdf.

[89] Baloch Z., Carayon P., Conte-Devolx B., Demers L.M., Feldt-Rasmussen U., Henry J.F., LiVosli V.A., Niccoli-Sire P., John R., Ruf J. (2003). Laboratory medicine practice guidelines. Laboratory support for the diagnosis and monitoring of thyroid disease. Thyroid.

[90] Padoan A., Clerico A., Zaninotto M., Trenti T., Tozzoli R., Aloe R., Alfano A., Rizzardi S., Dittadi R., Migliardi M. (2020). Percentile transformation and recalibration functions allow harmonization of thyroid-stimulating hormone (TSH) immunoassay results. Clin. Chem. Lab. Med..

[91] Mankarious S., Lee M., Fischer S., Pyun K.H., Ochs H.D., Oxelius V.A., Wedgwood R.J. (1988). The half-lives of IgG subclasses and specific antibodies in patients with primary immunodeficiency who are receiving intravenously administered immunoglobulin. J. Lab. Clin. Med..

[92] Sakai H., Fukuda G., Suzuki N., Watanabe C., Odawara M. (2009). Falsely elevated thyroid-stimulating hormone (TSH) level due to macro-TSH. Endocr. J..

[93] Shimatsu A., Hattori N. (2012). Macroprolactinemia: Diagnostic, clinical, and pathogenic significance. Clin. Dev. Immunol..

[94] Sheikhi V., Heidari Z. (2021). Increase in Thyrotropin Is Associated with an Increase in Serum Prolactin in Euthyroid Subjects and Patients with Subclinical Hypothyroidism. Med. J. Islam. Repub. Iran.

[95] Elenkova A., Petrossians P., Zacharieva S., Beckers A. (2016). High prevalence of autoimmune thyroid diseases in patients with prolactinomas: A cross-sectional retrospective study in a single tertiary referral centre. Ann. Endocrinol..

[96] Onal E.D., Saglam F., Sacikara M., Ersoy R., Cakir B. (2014). Thyroid autoimmunity in patients with hyperprolactinemia: An observational study. Arq. Bras. Endocrinol. Metabol..

[97] Hekimsoy Z., Kafesçiler S., Güçlü F., Ozmen B. (2010). The prevalence of hyperprolactinaemia in overt and subclinical hypothyroidism. Endocr. J..

[98] Kadoya M., Koyama S., Morimoto A., Miyoshi A., Kakutani M., Hamamoto K., Kurajoh M., Shoji T., Moriwaki Y., Koshiba M. (2017). Serum Macro TSH Level is Associated with Sleep Quality in Patients with Cardiovascular Risks—HSCAA Study. Sci. Rep..

[99] Reily C., Stewart T.J., Renfrow M.B., Novak J. (2019). Glycosylation in health and disease. Nat. Rev. Nephrol..

[100] Fiorentino T.V., Succurro E., Arturi F., Giancotti A., Peronace C., Quirino A., Sesti F., Andreozzi F., Hribal M.L., Perticone F. (2018). Serum IgG2 levels are specifically associated with whole-body insulin-mediated glucose disposal in non-diabetic offspring of type 2 diabetic individuals: A cross-sectional study. Sci. Rep..

[101] Gulcelik N.E., Usman A. (2010). Macroprolactinaemia in diabetic patients. Neuro Endocrinol. Lett..

[102] Hattori N., Aisaka K., Yamada A., Matsuda T., Shimatsu A. (2022). Prevalence and pathogenesis of macro-TSH in neonates: Analysis of umbilical cord blood from 939 neonates and their mothers. Thyroid.

[103] McCarthy A., Moran C. (2022). A grave interference: TSH interference due to macro-TSH post-thyroidectomy for graves” disease. Endocr. Abstr..

[104] Ohba K. (2021). An Update on the Pathophysiology and Diagnosis of Inappropriate Secretion of Thyroid-Stimulating Hormone. Int. J. Mol. Sci..

